# Supplementary material for: dia-PASEF data analysis using FragPipe and DIA-NN for deep proteomics of low sample amounts
Source: Nat Commun. 2022 Jul 8;13:3944. doi: 10.1038/s41467-022-31492-0 (PMC9270362; doi:10.1038/s41467-022-31492-0)
Supplement: Supplementary file 1 — Supplementary Information [file 41467_2022_31492_MOESM1_ESM.pdf]

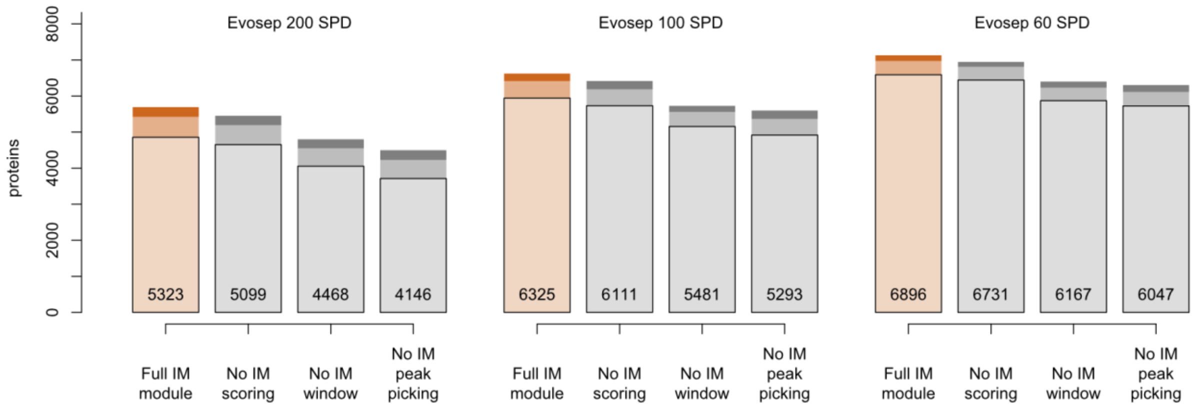

Supplementary Figure S1. **The impact of the algorithms of a software TIMS module on dia-PASEF performance**. The numbers of proteins quantified in Evosep One runs with different gradient lengths are compared between the fully functional module ('Full IM module'), the IM module without ion mobility scores that are calculated for each candidate peak group and are used by neural network-based q-value calculation ('No IM scoring'), with ion mobility windowed search further disabled ('No IM window') and with 2D-peak-picking further disabled ('No IM peak picking'). Numbers of proteins identified in 1, 2 or all 3 replicates are shown with different color shades; average numbers are indicated.

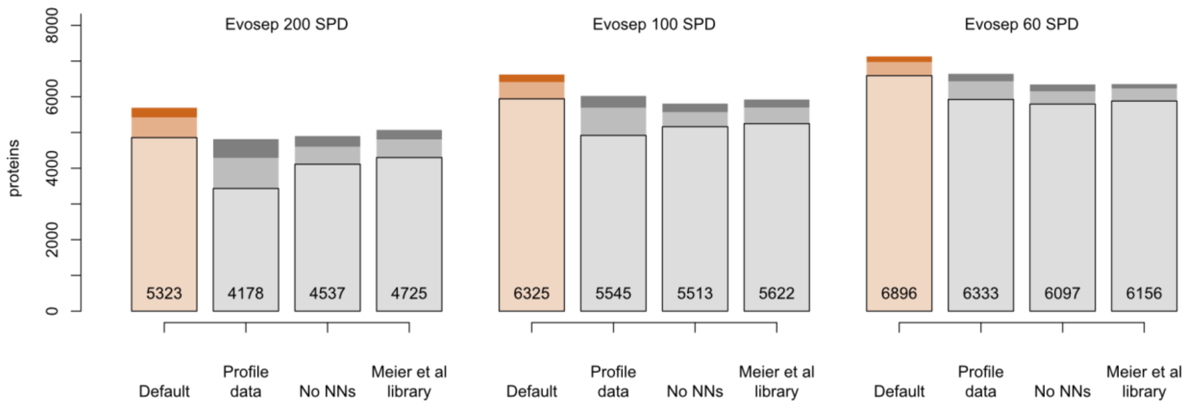

Supplementary Figure S2. **The impact of different processing approaches on dia-PASEF performance**. The numbers of proteins quantified in Evosep One runs with different gradient lengths are compared between the suggested processing approach (FragPipe + DIA-NN pipeline, 'Default'), same with DIA-NN using profile data for chromatogram extraction, with neural network classifier disabled in DIA-NN ('No NNs'), that is a linear classifier used instead, and with the spectral library described previously by Meier et al[1]. Numbers of proteins identified in 1, 2 or all 3 replicates are shown with different color shades; average numbers are indicated.

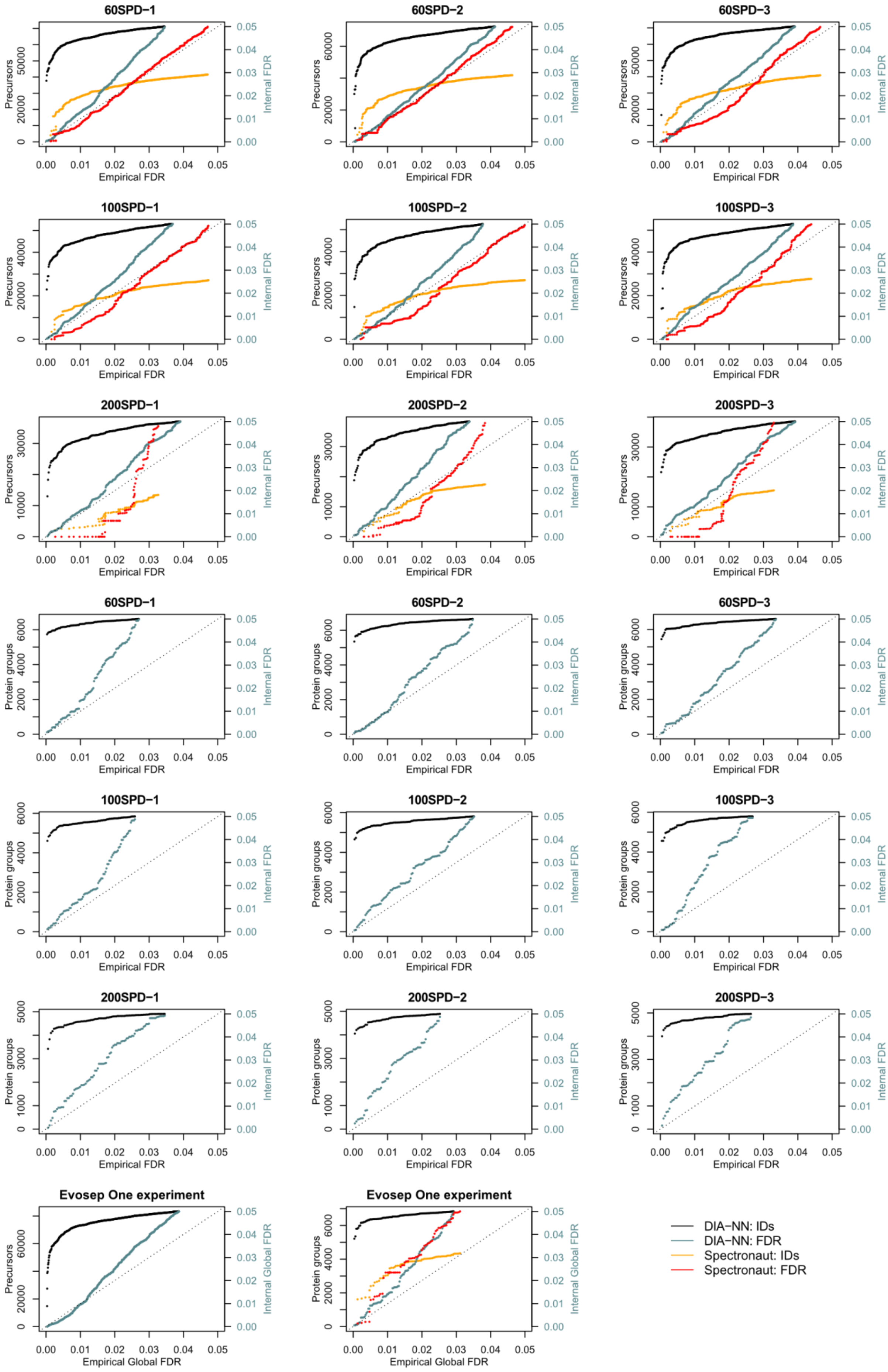

60SPD−1
60SPD−2
60SPD−3
100SPD−1
100SPD−2
100SPD−3
200SPD−1
200SPD−2
200SPD−3
60SPD−1
60SPD−2
60SPD−3
100SPD−1
100SPD−2
100SPD−3
200SPD−1
200SPD−2
200SPD−3
Evosep One experiment
Evosep One experiment
Precursors
Protein groups
Empirical FDR
Empirical Global FDR
Internal FDR
Internal Global FDR
DIA−NN: IDs
DIA−NN: FDR
Spectronaut: IDs
Spectronaut: FDR

Supplementary Figure S3. **False-discovery rate validation**. Evosep One acquisitions at different throughputs (60 SPD - 200 SPD) were analyzed. Precursor and protein numbers (black) as well as software-reported FDR values (grey) were plotted against the FDR estimates experimentally obtained (Methods) using a two-species human-*A.thaliana* spectral library[2], for individual runs (run-specific FDR) as well as for the entire experiment (Global FDR). Identifications were ordered by their q-values and visualization was performed for precursors and proteins starting with rank 1000. Each point corresponds to an *A. thaliana* precursor or protein called in a human sample. The benchmark reveals that FDR values reported by DIA-NN operating the new TIMS module are highly conservative. Further, the benchmark demonstrates that the software can achieve < 0.1% false discovery rates, at both precursor and protein level, regardless of the data complexity (gradient length). Performance of Spectronaut on the same data is plotted for comparison. Spectronaut does not report run-specific protein q-values, hence it was not included in the respective benchmarks.

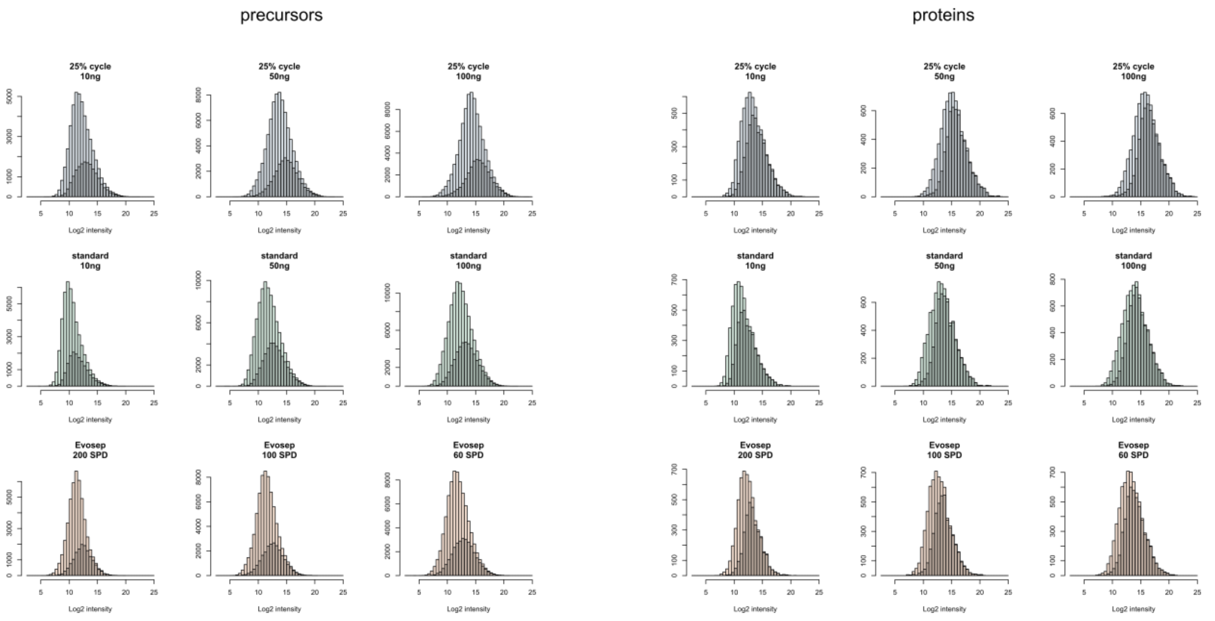

Supplementary Figure S4. **Identification overlap for precursors and proteins**. Distribution of precursor (left) and protein (right) intensities is shown. Identifications shared with the original dia-PASEF workflow[1] are shaded in grey. Our software workflow gains in the identification of medium- and low-abundant precursors and proteins.

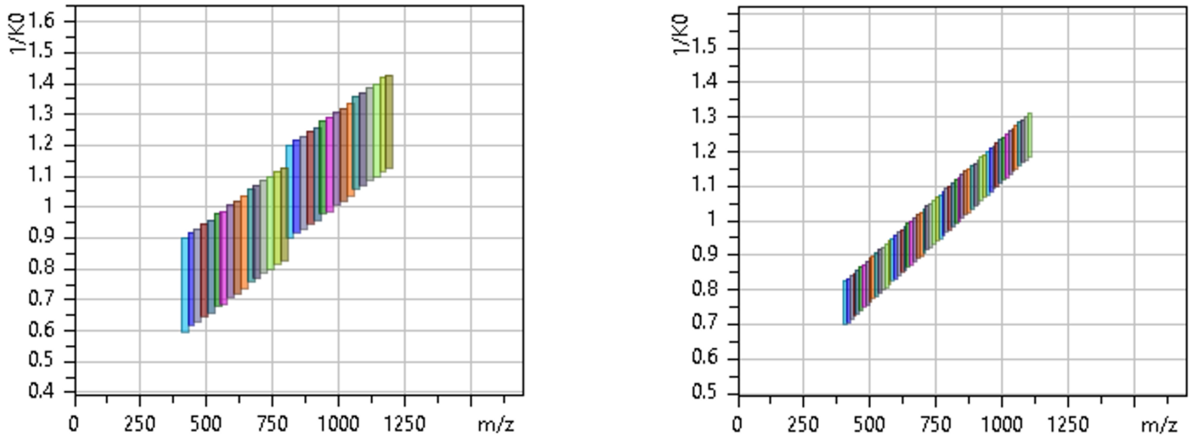

Supplementary Figure S5. **dia-PASEF acquisition schemes on Bruker timsTOF Pro 2**. Left: acquisition scheme used for injections higher than 50ng of peptides. Right: acquisition scheme used for injections of 50ng or less peptides. Scheme visualization was performed using the Bruker timsControl software.

References

1. Meier, F. *et al.* diaPASEF: parallel accumulation–serial fragmentation combined with data-independent acquisition. *Nat. Methods* **17**, 1229–1236 (2020).

2. Muntel, J. *et al.* Surpassing 10000 identified and quantified proteins in a single run by optimizing current LC-MS instrumentation and data analysis strategy. *Molecular Omics* **15**, 348–360 (2019).
