# Supplementary material for: dia-PASEF data analysis using FragPipe and DIA-NN for deep proteomics of low sample amounts
Source: Nat Commun. 2022 Jul 8;13:3944. doi: 10.1038/s41467-022-31492-0 (PMC9270362; doi:10.1038/s41467-022-31492-0)
Supplement: Supplementary file 2 — Reporting Summary [file 41467_2022_31492_MOESM2_ESM.pdf]

nature portfolio

Corresponding author(s): Markus Ralser

Last updated by author(s): Jun 3, 2022

Reporting Summary

Nature Portfolio wishes to improve the reproducibility of the work that we publish. This form provides structure for consistency and transparency in reporting. For further information on Nature Portfolio policies, see our Editorial Policies and the Editorial Policy Checklist.

Statistics

For all statistical analyses, confirm that the following items are present in the figure legend, table legend, main text, or Methods section.

n/a	Confirmed	
☐	☒	The exact sample size (*n*) for each experimental group/condition, given as a discrete number and unit of measurement
☒	☐	A statement on whether measurements were taken from distinct samples or whether the same sample was measured repeatedly
☒	☐	The statistical test(s) used AND whether they are one- or two-sided<br>*Only common tests should be described solely by name; describe more complex techniques in the Methods section.*
☒	☐	A description of all covariates tested
☒	☐	A description of any assumptions or corrections, such as tests of normality and adjustment for multiple comparisons
☐	☒	A full description of the statistical parameters including central tendency (e.g. means) or other basic estimates (e.g. regression coefficient) AND variation (e.g. standard deviation) or associated estimates of uncertainty (e.g. confidence intervals)
☒	☐	For null hypothesis testing, the test statistic (e.g. *F*, *t*, *r*) with confidence intervals, effect sizes, degrees of freedom and *P* value noted<br>*Give P values as exact values whenever suitable.*
☒	☐	For Bayesian analysis, information on the choice of priors and Markov chain Monte Carlo settings
☒	☐	For hierarchical and complex designs, identification of the appropriate level for tests and full reporting of outcomes
☒	☐	Estimates of effect sizes (e.g. Cohen's *d*, Pearson's *r*), indicating how they were calculated

Our web collection on statistics for biologists contains articles on many of the points above.

Software and code

Policy information about availability of computer code

Data collection	HeLa dilution series data were collected using Bruker timsControl 2.0.53
Data analysis	The following software was used for data analysis: DIA-NN 1.8.1, FragPipe 15, MSFragger 3.2, Philosopher 3.4.13, Easypqp 0.1.9, Spectronaut 14.3.200701.47784, R version 3.6.0

For manuscripts utilizing custom algorithms or software that are central to the research but not yet described in published literature, software must be made available to editors and reviewers. We strongly encourage code deposition in a community repository (e.g. GitHub). See the Nature Portfolio guidelines for submitting code & software for further information.

Data

Policy information about availability of data

All manuscripts must include a data availability statement. This statement should provide the following information, where applicable:

- Accession codes, unique identifiers, or web links for publicly available datasets
- A description of any restrictions on data availability
- For clinical datasets or third party data, please ensure that the statement adheres to our policy

The mass spectrometry proteomics data (HeLa dilution series) have been deposited to the ProteomeXchange Consortium (http://proteomecentral.proteomexchange.org) via the PRIDE partner repository35 with the dataset identifier PXD029836.

Previously generated data used in this study is further available from ProteomeXchange Consortium repositories with identifiers PXD017703 [http://proteomecentral.proteomexchange.org/cgi/GetDataset?ID=PXD017703], PXD022216 [http://proteomecentral.proteomexchange.org/cgi/GetDataset?

March 2021

ID=PXD022216] and PXD013658 [http://proteomecentral.proteomexchange.org/cgi/GetDataset?ID=PXD013658]. The UP000005640 [https://www.uniprot.org/proteomes/UP000005640] (human) and UP000002311 [https://www.uniprot.org/proteomes/ UP000002311] (yeast) sequence databased used in this work are available from the UniProt repository.

Software output reports, spectral libraries, PSM tables, logs and the pipeline configuration file were deposited to an OSF (Open Science Framework) repository https://doi.org/10.17605/OSF.IO/8EPQH.

Field-specific reporting

Please select the one below that is the best fit for your research. If you are not sure, read the appropriate sections before making your selection.

- [x] Life sciences
- [ ] Behavioural & social sciences
- [ ] Ecological, evolutionary & environmental sciences

For a reference copy of the document with all sections, see nature.com/documents/nr-reporting-summary-flat.pdf

Life sciences study design

All studies must disclose on these points even when the disclosure is negative.

Sample size	Sample size calculation was not performed, as sample size was not considered important for the software benchmarks described in the manuscript, due to low variation in identification numbers across mass spectrometry runs acquired with the same settings. Therefore, a single replicate of each injection amount was acquired on timsTOF Pro 2. In all other cases, the sample size was determined by the numbers of mass spectrometry acquisitions available in the respective public datasets.
Data exclusions	No data were excluded.
Replication	All replication attempts (3 total) for our data analysis pipeline were successful and yielded identical results. The timsTOF Pro 2 experiment was not replicated, as the results obtained showed satisfactory performance, which was the purpose of this technical benchmark.
Randomization	Randomization is not applicable to the technical benchmarks described.
Blinding	Blinding is not applicable to the technical benchmarks described.

Reporting for specific materials, systems and methods

We require information from authors about some types of materials, experimental systems and methods used in many studies. Here, indicate whether each material, system or method listed is relevant to your study. If you are not sure if a list item applies to your research, read the appropriate section before selecting a response.

Materials & experimental systems

n/a	Involved in the study	
x		Antibodies
	x	Eukaryotic cell lines
x		Palaeontology and archaeology
x		Animals and other organisms
x		Human research participants
x		Clinical data
x		Dual use research of concern

Methods

n/a	Involved in the study	
x		ChIP-seq
x		Flow cytometry
x		MRI-based neuroimaging

Eukaryotic cell lines

Policy information about cell lines

Cell line source(s)	HeLa (ATCC) cells were used
Authentication	None of the cell lines were authenticated
Mycoplasma contamination	Cells were negative for mycoplasma contamination
Commonly misidentified lines (See ICLAC register)	No commonly misidentified lines were used in the study
